# Microbial diagenesis of dissolved organic matter from the ocean’s surface to abyssal depths: a case study in the Humboldt upwelling system

**DOI:** 10.3389/fmicb.2025.1677097

**Published:** 2025-12-16

**Authors:** Anja Engel, Benjamin Pontiller, Kevin W. Becker, Chie Amano, Zihao Zhao, Gerhard J. Herndl, Cindy Lee

**Affiliations:** 1 GEOMAR Helmholtz Centre for Ocean Research Kiel, Kiel, Germany; 2 Kiel University, Kiel, Germany; 3 Department of Functional and Evolutionary Ecology, Microbial Oceanography Working Group, University of Vienna, Vienna, Austria; 4 School of Marine and Atmospheric Sciences, Stony Brook University, Stony Brook, NY, United States

**Keywords:** dissolved organic matter, deep ocean, amino acids, microbial activity, Humboldt upwelling system

## Abstract

Marine dissolved organic matter (DOM) represents one of Earth’s largest dynamic carbon pools—comparable in scale to atmospheric CO₂. Primarily derived from phytoplankton production in the sunlit surface ocean, DOM serves as a key substrate for heterotrophic microbes that actively transform and recycle it. The portion remaining after microbial diagenesis contributes to the long-lived deep-sea reservoir of refractory dissolved organic carbon (RDOC) with turnover times up to millennia. DOC lability is an important trait determining microbial utilization as well as carbon storage time in the ocean and can be inferred from its chemical composition, particularly changes in individual amino acids (AAs). In this study, we examined dissolved (DOC) and particulate organic carbon (POC) distribution, composition and concentration of dissolved hydrolyzable AAs (DHAA), microbial community structure, and activity along depth profiles from the surface to the abyssopelagic zone (down to 5,000 m) in the Humboldt upwelling system off Chile—one of the ocean’s most productive regions. Our results show a pronounced decrease in DOC concentration and lability, and in viral and prokaryotic abundance with depth. Below the mesopelagic zone, DOC displayed characteristics of RDOC: <42 μmol C L^−1^, [DHAA-C]:[DOC] ~ 0.6%, and a glycine fraction of ~75 mol% DHAA. Bacterial biomass production and extracellular enzyme activities (EEA), however, were detectable below the mesopelagic zone and even at abyssal depths, albeit at very low rates. Cell-specific EEA and the proportion of high nucleic acid (HNA) cells increased with depth suggesting adaptation to an extremely low-substrate environment. We discuss microbial carbon turnover under varying assumptions of bacterial growth efficiency and conclude that microbial life in the bathy- and abyssopelagic zones of the Humboldt Current is likely sustained by the flux of sinking particulate organic matter.

## 1 Introduction

With 662 Pg C, marine dissolved organic matter (DOM) is one of Earth’s major carbon reservoirs, comparable to the size of the atmospheric CO_2_ reservoir and about 200 times greater than the inventory of marine biomass (Hansell et al., 2009). The primary source of marine DOM is phytoplankton in the sunlit surface layer that forms the nutritional basis for heterotrophic microorganisms that consume, respire, modify, and again release DOM. Approximately one-quarter of the primary produced DOM is rapidly recycled (Moran et al., 2022), and the remaining accumulates in the surface ocean for up to 1.5 years (Hansell, 2013). A small portion remains after microbial degradation, and contributes to the bulk of the oceanic dissolved organic carbon (DOC) pool with lifetimes up to the order of millennia, making it a major sink of CO_2_ initially fixed in the surface ocean (Lechtenfeld et al., 2015; Jiao et al., 2014). The size of the reservoir, along with its function as a source of substrate for microbial heterotrophs and as a sink for autotrophically fixed CO_2_, implies a central role for DOC in ocean carbon cycling (Hansell, 2013; Hansell and Carlson, 2014). DOC export to great depths (>1,000 m) primarily occurs via the meridional overturning circulation, and consequently, the oldest DOC has been observed in the North Pacific (Hansell, 2013), as water masses in this region are not locally formed. Other export mechanisms include winter convective mixing and the solubilization of sinking particulate organic matter (POM) during export (Hansell and Orellana, 2021). As a consequence of production, reworking and decomposition, marine DOM exists along a continuum of lifetimes (Amon and Benner, 1996). Based on its microbial turnover time, DOM lability has been defined (Davis and Benner, 2007): Labile DOC (LDOC) is freshly produced by organisms, mainly algae, and is directly accessible to microbes, resulting in a short lifetime of hours to weeks. Semi-labile DOC (SLDOC) resists rapid microbial turnover, allowing it to accumulate during the productive season, with lifetimes ranging from months to decades. Refractory dissolved organic carbon (RDOC) can persist in the ocean for centuries to millennia. However, the mechanisms underlying RDOC long-term stability and potential for microbial utilization—thereby acting as a possible CO₂ source—remain under debate (Jiao et al., 2010; Baltar et al., 2021; Dittmar et al., 2021). Limited data on DOC lability and microbial activity in the deep sea continue to constrain our understanding of deep ocean microbial carbon cycling.

DOC lability can be inferred from its chemical composition. DOM freshly produced by phytoplankton is largely composed of known cellular building blocks: polysaccharides, proteins, lipids and DNA. During microbial decomposition, this DOM is molecularly diversified, and refractory compounds accumulate over time, making up the majority of deep-sea DOM. While a large fraction of deep-sea DOM is uncharacterized, molecular-level analysis of the characterizable fraction is the key understanding DOM dynamics in the ocean (Benner, 2002; Davis and Benner, 2007). In particular, the relative abundance of individual amino acids (AAs) is altered during decomposition, and AA composition has been used as a biomarker for microbial diagenesis of organic matter (e.g., Dauwe and Middelburg, 1998; Dauwe et al., 1999; Yamashita and Tanoue, 2003). AAs, primarily found in proteins, are key components of both living biomass and non-living organic material within the ocean’s particulate and dissolved organic matter pools. Their uptake and transformation by heterotrophic organisms result in distinct alterations in AA composition, making AA-based biogeochemical indicators valuable tools for understanding organic matter cycling and degradation processes (Wakeham and Lee, 1993; Cowie and Hedges, 1994). Specific markers, such as D-amino acids and non-protein AAs, provide insights into microbial processing and the formation of recalcitrant DOM (Lee et al., 2000; Duan and Bianchi, 2007; Davis et al., 2009). Dissolved free amino acids (DFAA) are considered among the most labile compounds and are preferentially degraded by heterotrophic microbial communities (Jorgensen et al., 1993). Glycine (Gly) is an essential amino acid and, together with glutamic acid, alanine, and aspartic acid, one of the predominant DFAA in seawater. Dissolved hydrolyzable amino acids (DHAA) have been classified as semi-labile DOM (e.g., Fuhrman, 1987; Amon et al., 2001; Davis et al., 2009). Although free, i.e., monomeric, Gly is highly labile, relative glycine (Gly) content of DHAA typically indicates more refractory organic matter, while a high leucine (Leu) content indicates higher lability of DHAA and organic matter in general (Amon et al., 2001; Dauwe and Middelburg, 1998; Kaiser and Benner, 2009). The non-protein AA y-aminobutyric acid (GABA) is a degradation product of glutamic acid and has been widely used as an indicator for more refractory organic matter. AAs thus serve as valuable biomarkers for understanding the microbial transformation of DOM in the ocean. Their composition can help trace the biological origin of DOM, distinguish between algal and microbial sources, and assess the degree of degradation and microbial reworking. This makes AAs essential tools for linking microbial metabolism to long-term carbon storage in marine systems. Knowledge of DOC lability and DHAA composition in the deep ocean, particularly below mesopelagic depths, is still limited.

Coastal upwelling systems like the Humboldt current system, where nutrient-rich waters of the deep ocean reach the sunlit surface ocean and promote high primary production, are hotspots of organic matter cycling (Engel et al., 2022; Romera-Castillo et al., 2016; Delgadillo-Nuño et al., 2024) and microbial diagenesis of DOM (Loginova et al., 2019; Maßmig and Engel, 2021). Investigating diagenetic changes in DOM composition from the surface to the deep ocean in upwelling systems may help to better understand microbial alterations of organic matter and their contribution to carbon storage in the ocean, i.e., the microbial carbon pump. Here, we present changes in the composition and concentration of organic matter, along with the microbial communities and their activity, in samples from the surface to the abyssopelagic zone in the Humboldt upwelling system off the coast of Chile, one of the most productive regions in the ocean.

## 2 Methods

### 2.1 Study area and sample collection

**Figure 1:**
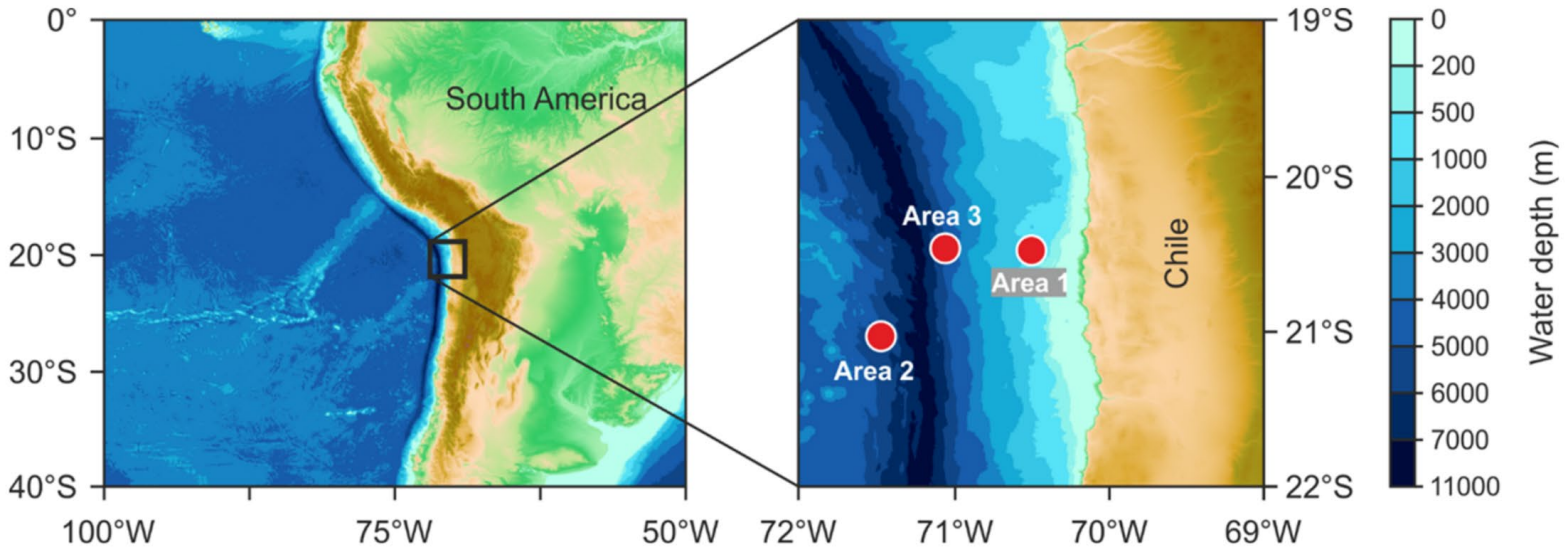
Overview of the three working areas in the Humboldt Current off Chile sampled during RV SONNE expedition HOMER (SO288). The gridded bathymetry map was obtained from http://www.gebco.net (General Bathymetric Chart of the Oceans, GEBCO 2024 Grid).

Sea water samples were collected during the RV SONNE cruise SO288 (15 January 2022–15 February 2022) within the framework of the HOMER project in the Southeast Pacific off Chile (Figure 1). At three stations, water samples were collected at 12 depths throughout the water column using standard 12 L Niskin-type bottles attached to a CTD rosette and represented the different pelagic zones: 25 m and 100 m (epipelagic), 250, 500, 750 and 1,000 m (mesopelagic), 1,500-3800 m (bathypelagic; here different depths were sampled due to differences in bathymetry), and 4,000, 4,500 and 5,000 m (abyssopelagic). Temperature, oxygen, and pressure were measured using a Sea-Bird SBE 9-plus CTD system (Sea-Bird Electronics, Inc.), and chlorophyll *a* (Chl *a*) concentrations were detected using a WETStar fluorometer (WET Labs).

### 2.2 Chemical analysis

#### 2.2.1 Dissolved nutrient analysis

Samples (10 mL) were collected in triplicate in plastic tubes and stored at −20 °C until analysis. Prior to analysis, the samples were filtered using pre-washed (10% HCl) cellulose acetate filters (pore size 0.45 μm). Dissolved nutrients (NO_3_^−^, NO_2_^−^, SiO_4_^−^ and PO_4_^3−^) were measured using a Quaatro Seal Autoanalyser. The limit of detection for NO_3_^−^, NO_2_^−^, SiO_4_^−^ and PO_4_^3−^ were 0.22 μmol L^−1^, 0.004 μmol L^−1^, 0.34 μmol L^−1^ and 0.017 μmol L^−1^, respectively.

#### 2.2.2 Particulate organic carbon and nitrogen

For the analyses of particulate organic carbon (POC) and nitrogen (PON), water samples (1 L) were filtered onto pre − combusted (450 °C for 5 h) Whatman GF/F filters (25 mm, 0.7 μm) under low pressure (<200 mbar). Filters were stored frozen at −20 °C until analysis. Prior to analysis, filters were acid−fumed (37% HCl for 24 h) in order to remove inorganic carbon and dried at 40 °C for 24 h. Subsequently, filters were wrapped in tin cups (8 × 8 × 15 mm) and analyzed according to Sharp (Sharp, 1974) using a EuroEA Elemental Analyzer (HEKAtech, Wegberg, Germany). Blank filters (POC: 0.69 ± 0.16 μmol per filter; PON: 0.066 ± 0.050 μmol per filter) were used to correct for potential contaminations.

#### 2.2.3 Dissolved organic carbon

Prior to every sampling for dissolved organic carbon (DOC), a syringe was rinsed with 50 mL MilliQ water. Subsequently, a glass micro fibre (GMF) filter with a pore size of 0.45 μm was attached to the syringe and rinsed with 50 mL of the respective seawater sample. Finally, 20 mL of the seawater sample was filtered through the rinsed GMF filter into a pre-combusted (450 °C for 5 h) glass ampule, and 20 μL of 30% hydrochloric acid (Suprapur®, HCl) was added. The ampules were sealed with a gas burner and stored at 4C until analysis. The samples were measured with a high-temperature catalytic oxidation TOC analyzer (TOC-VCSH, Shimadzu), according to Engel and Galgani (2016).

#### 2.2.4 Dissolved hydrolyzable amino acids

Samples for dissolved hydrolyzable AAs (DHAA) were collected by filtering 4 mL in duplicate through 0.45 μm pore size filters pre-rinsed with MilliQ water. The samples were stored frozen at −20° C until analysis according to Lindroth and Mopper (1979) and Dittmar et al. (2009) with some modifications. Before analysis, an aliquot of the samples (1 mL) was acid hydrolyzed with 12 M HCl for 24 h at 100 °C with the addition of an antioxidant [11 mM ascorbic acid; Dittmar et al. (2009)]. HCl was removed using N_2_ under vacuum in a microwave at 60 °C. To ensure complete removal of HCl, 1 mL of MilliQ water was added twice, and the sample was redried after each addition. Finally, the sample was dissolved in 950 μL MilliQ water, and 50 μL borate buffer was added to adjust the pH to 9.5. After automated in-line derivatization with o-phthaldialdehyde and mercaptoethanol, the samples were analyzed using an Agilent 1,260 Infinity high-performance liquid chromatography (HPLC) system with fluorescence detection. Separation of thirteen different AAs was achieved by reversed-phase HPLC on a C_18_ column (Phenomenex Kinetex, 2.6 μm, 150 × 4.6 mm). A linear gradient was run with 100% Solvent A [5% acetonitrile (LiChrosolv, Merck, HPLC gradient grade) in sodiumdihydrogenphospate buffer (pH 7.0; Merck, Suprapur)] to 22% solvent B (acetonitrile) for 50 min. Fluorescence detection was carried out at excitation and emission wavelengths of 470 and 530 nm, respectively. The following standards were analyzed at different concentrations and used for quantification: Asparagine + aspartic acid (AsX), glutamine + glutamic acid (GlX), serine (Ser), glycine (Gly), threonine (Thr), arginine (Arg), alanine (Ala), tyrosine (Tyr), valine (Val), isoleucine (Ile), phenylalanine (Phe), leucine (Leu), and *γ*- aminobutyric acid (GABA). Procedural blanks were analyzed and used to correct concentrations in the samples.

To determine the relative degradation state of DOM, the AA degradation index (DI) was calculated according to Dauwe et al. (1999). The AAs used were AsX, GlX, Ser, Thr, Gly, Arg, Ala, Tyr, Val, Ile, Phe, Leu, and GABA. For the calculation of DI from DHAA in this study, mole percentages of AAs were standardized using averages and standard deviations, and multiplied by factor coefficients based on principal component analysis (Supplementary Figure S1).

### 2.3 Prokaryotic abundance and activity

#### 2.3.1 Flow cytometry

Seawater (1.7 mL) was fixed with glutaraldehyde (1.2% final conc.) to determine prokaryotic cell abundance. Cryovials were flash-frozen using liquid N_2_ and stored at −80 °C until analysis. The flow cytometer (FACSCalibur, Becton Dickinson, USA) was calibrated and standardized with TruCount beads (Becton Dickinson, USA). Since the flow cytometer’s detection limit was < 50 μm, we filtered the samples through a mesh before counting using Cell Quest 3.3 software with a DL of 2000 events s^−1^. To detect prokaryotic cells, the filtered samples were stained with SybrGreenI (Thermo Fisher Scientific, USA), and signals from autofluorescence were excluded. Subpopulations of low nucleic acid content (LNA) and high nucleic acid content (HNA) bacteria were derived by distinguishing between differences in fluorescence intensity (Bouvier et al., 2007; Robertson and Button, 1989; Sherr et al., 2006). For abundance of viruses, samples were preserved with glutaraldehyde (0.5% final conc.) until analysis following Marie et al. (1999).

#### 2.3.2 Heterotrophic bacterial biomass production

Heterotrophic bacterial biomass production (BBP) was measured onboard the research vessel based on the filtration method (Kirchman, 2001). In brief, duplicates and one killed control of each 540 mL sample were spiked with ^3^H-leucine (BioTrend, USA; specific activity: 100 Ci mmol) at a final concentration of 20 nmol L^−1^ (≤100 m) and 5 nmol L^−1^ (>100 m) at all stations. The samples were incubated for 125 h at *in situ* temperature ± 1 °C in the dark and terminated using formaldehyde (2% final concentration). Disintegrations per minute were counted on board with a liquid scintillation counter (Tri-Carb 2,910 TR, PerkinElmer). To convert the leucine incorporation into BBP, we applied a factor of 1.5 kg C mol leucine^−1^ and assumed no intracellular isotope dilution (Simon and Azam, 1989). BBP was not measured in oxygen-depleted waters (~0 μmol L^−1^) to avoid potential artifacts caused by changes in oxygen concentration during sampling and incubation, which could bias production estimates.

#### 2.3.3 Extracellular enzymatic activity (EEA)

Potential hydrolysis rates were determined with fluorogenic substrate analogues: 4-methylcoumarinyl-7-amide (MCA)-L-Leucin-7-amido-4-methylcoumarin, (leucine aminopeptidase, LAPase), 4-Methylumbelliferyl (MUF)-*β*-D-glucopyranoside (β-glucosidase, BGase), 4-Methylumbelliferyl-N-acetyl-β-D-glucosaminide (N-acetyl-glucosaminidase, NAG), and 4-Methylumbelliferyl-phosphate (alkaline phosphatase, APase) according to Hoppe (1983). EEA measurements were conducted as previously described (Baltar et al., 2016). In brief, EEA was measured immediately after sampling and substrate addition. Triplicate samples (290 μL) were incubated in black 96 well plates (Costar), in the dark, and at *in situ* temperature for 12–24 h, and fluorescence was measured using a spectrofluorometer (FLUOstar Optima, BMG Labtech) at an excitation of 355 nm and an emission of 460 nm. Bulk (unfiltered) and filtered (0.2 μm) subsamples from all depth layers of the water column, without substrate additions, were used to determine the background fluorescence of the seawater. Fluorescence values obtained at the beginning and the final reading were corrected for the corresponding seawater blanks, and the fluorescence increase over time was converted into hydrolysis rates using standard curves with different final concentrations (1.25, 2.5, 10, 50, 100, 500 and 1,000 nmol L^−1^) of MCA and MUF fluorochromes (Sigma Aldrich) in triplicates and additions of 290 μL pooled and pre-filtered (0.2 μm) seawater. The following final substrate concentrations, previously determined as saturating substrate concentrations (Baltar et al., 2016), were used: 30 μmol L^−1^ for BGase and NAG, 100 μmol L^−1^ for APase, and 500 μmol L^−1^ for LAPase. Bulk enzymatic activity refers to the total enzymatic activity from the particle-attached and free-living microbial community within the sampled water and is measured in terms of substrate utilization per unit of time (hour). Cell-specific enzyme activity rates refer to bulk enzymatic activities normalized to the total cell counts using flow cytometry.

#### 2.3.4 Microbial community composition

Water samples for microbiome analysis were collected at the depths of 25, 1,500, and 2,500 m from area 1, 2000, 2,500, and 3,000 m from area 2, and 100, 750, and 1,000 m from area 3 using 12 L Niskin bottles. For each discrete water sample, approximately 100 L of water was filtered through 142 mm PC filters (Whatman) with a filter pore size of 3.0 and 0.22 μm (3–0.22 μm). The filters were immediately stored at −80 °C and shipped on dry ice until further processing. Total DNA was extracted from one-quarter of each filter using the PowerWater DNA extraction kit (Qiagen, Germany) according to the manufacturer’s instructions. DNA concentrations were evaluated using a Qubit 4.0 Fluorometer (Thermo Fisher Scientific, Waltham, MA, USA). For DNA library preparation, metagenomic high-throughput libraries were constructed using the DNA Library Prep Kit for Illumina. Sequencing was performed on a NovaSeq 6,000 platform (Illumina, San Diego, CA, USA) with a 2 × 150 bp paired-end run.

Raw reads were adapter-trimmed, filtered using AdaptorRemoval, and quality control was performed using FastQC with default parameters. Reads containing adaptors, low-quality reads (specify here), or unpaired high-quality reads were removed. The ribosomal RNA (rRNA) Illumina sequencing reads (miTAGs) were identified from the clean metagenomic reads using SortMeRNA to identify ribosomal RNA gene fragments from both forward and reverse metagenomic reads.

16S miTAGs were processed following the QIIME2 pipeline. They were then clustered into amplicon sequence variants (ASVs) with ≥97% similarity using the dada2 algorithm. The tag sequence with the highest abundance was selected as the representative sequence for each ASV. The taxonomic assignment of the 16S miTAG ASVs was conducted using the Feature-classifier classify-sklearn command in QIIME2 against the Silva (release 138) database.

## 3 Results and discussion

### 3.1 The physical and chemical environment

**Figure 2:**
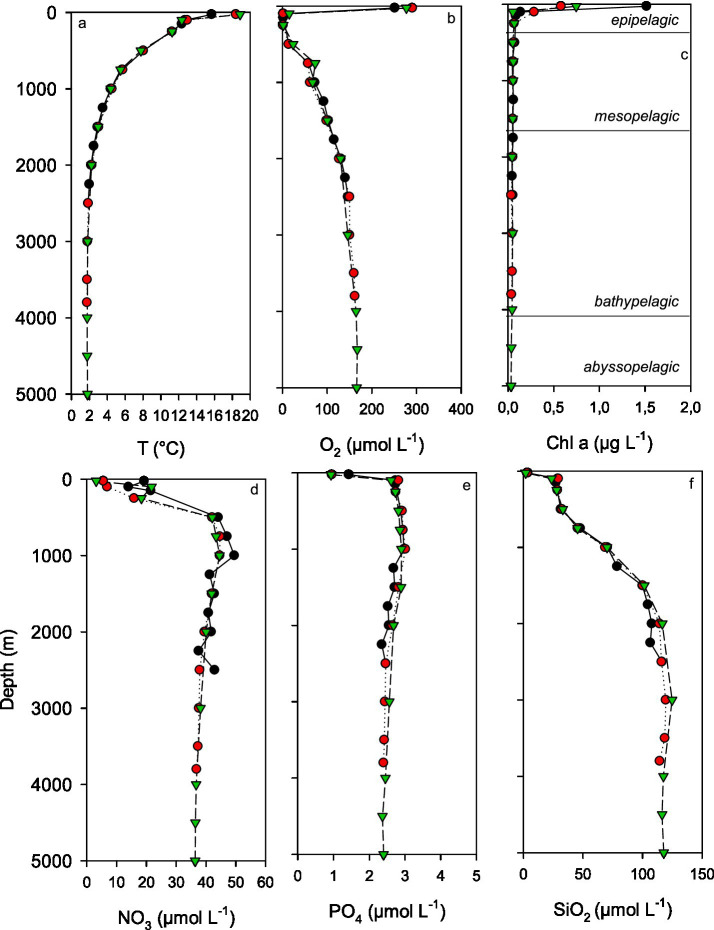
**(a–f)** Whole water column profiles of temperature (T, **a**), oxygen (O_2_, **b**), chlorophyll *a* (Chl *a*, **c**), and the inorganic nutrients [nitrate (NO_3_, **d**), phosphate (PO_4_, **e**), and silicate (SiO_2_, **f**)], for the three stations sampled in the Humboldt current off Chile during SO288. Symbols: black (area 1), red (area 2), green (area 3).

In our study area, three major water masses were identified based on temperature and salinity profiles: Subtropical Water (STW), Equatorial Subsurface Water (ESSW), and Pacific Deep Water (PDW) (Supplementary Figure S2). Water mass analysis of the three profiles showed slight variability at the surface, while forming a very homogeneous water mass in deeper layers. Temperature declined from surface values of 18.86–15.68 °C to ~1.7 °C at the deepest depths of 5,000 m (Figure 2a).

At each of the three stations, the oxygen profiles showed a pronounced oxygen minimum zone (OMZ) between 58 ± 10 and 833 ± 156 m (O_2_ < 5 μmol L^−1^) (Figure 2b), which is typical for the ESSW off Chile (Neshyba, 1979; Morales et al., 1999) and is generated by the combination of high oxygen demand during organic matter remineralization and the sluggish ventilation in the region (Engel et al., 2023). Oxygen concentrations ~0 μmol L^−1^, which is considered anoxic, occurred between 86 ± 25 and 442 ± 14 m. Oxygen concentrations below the OMZ, reached a maximum of 167 μmol L^−1^ below 4,000 m, as PDW gets re-oxygenated through mixing with oxygen-rich AAIW (Silva et al., 2009). The highest concentrations of Chl *a* in the epipelagic zone ranged from 0.58 μg L^−1^ to 1.52 μg L^−1^ and declined sharply with depth (Figure 2c), falling below the detection limit (0.06 μg L^−1^) beneath the epipelagic zone. Chl *a* concentration in the region varies strongly, due to seasonal differences and differences in upwelling intensity. Maximum Chl *a* concentration reported by Morales et al. (1996) was more than five times higher than our measured concentrations, despite the enhanced upwelling caused by La Niña conditions during our sampling. While sampling by Morales et al. (1996) took place during the Austral winter and spring, our sampling took place during the Austral summer. The concentrations of the inorganic nutrients nitrate, phosphate, and silicate showed the lowest values in the oxygenated surface waters (25 m), with concentrations declining from the coast toward the open sea: 19.22–3.12 μmol L^−1^ for nitrate, 1.43–0.93 μmol L^−1^ for phosphate, and 3.68–1.97 μmol L^−1^ for silicate (Figures 2d–f).

The higher surface nutrient concentrations at the more coastal station in area 1, together with the shallow upper OMZ boundary, indicated upwelling conditions likely due to a prominent and persistent ‘double-dip’ La Niña event that occurred at the time of sampling (Hasan et al., 2022). The highest nutrient concentrations in the meso- and bathypelagic waters were 49.5 μmol L^−1^ for nitrate, 9.37 μmol L^−1^ for phosphate and 125 μmol L^−1^ for silicate (Figures 2d–f). The nutrient profiles were very similar at the three stations. Since the focus of this study was on the fate of organic matter with depth and because the differences in physical and chemical environments were small between the sampling areas, we combined data from the three sampling areas for the same pelagic zone for the following analysis.

### 3.2 Changes in organic matter over depth

**Figure 3:**
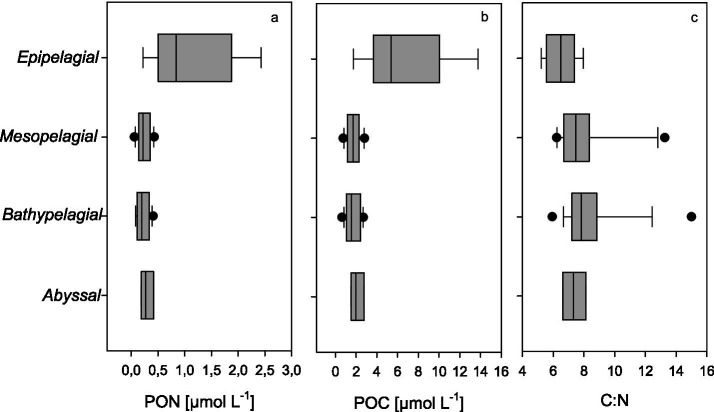
**(a–c)** Distribution of particulate organic matter in the water column off Chile. Particulate organic nitrogen (PON, **a**), particulate organic carbon (POC, **b**), and the molar ratio of POC: PON (C:N, **c**). Boxes here and in the following figures represent the interquartile ranges, the line within the box the median, and the whiskers extend to the minimum and maximum values within 1.5 times the interquartile range.

PON and POC were highest in the surface waters with 13.8 μmol L^−1^ and 2.43 μmol L^−1^, respectively (Figures 3a,b). POC concentrations decreased sharply below the epipelagic zone and remained almost unchanged down to the greatest depth. Profiles of POC and PON agreed well with previous observations off the Peruvian coast (Franz et al., 2012). The molar POC: PON ratio was close to the Redfield ratio (C:N = 6.6) of marine particles in surface samples and became more variable with depth, occasionally reaching values >10 in the mesopelagic (Figure 3c). This suggests that sinking particles were enriched in carbon either through preferential degradation of PON or due to higher amounts of carbon-rich compounds, such as transparent exopolymer particles (TEP), which are often found in marine snow (Alldredge et al., 1993).

**Figure 4:**
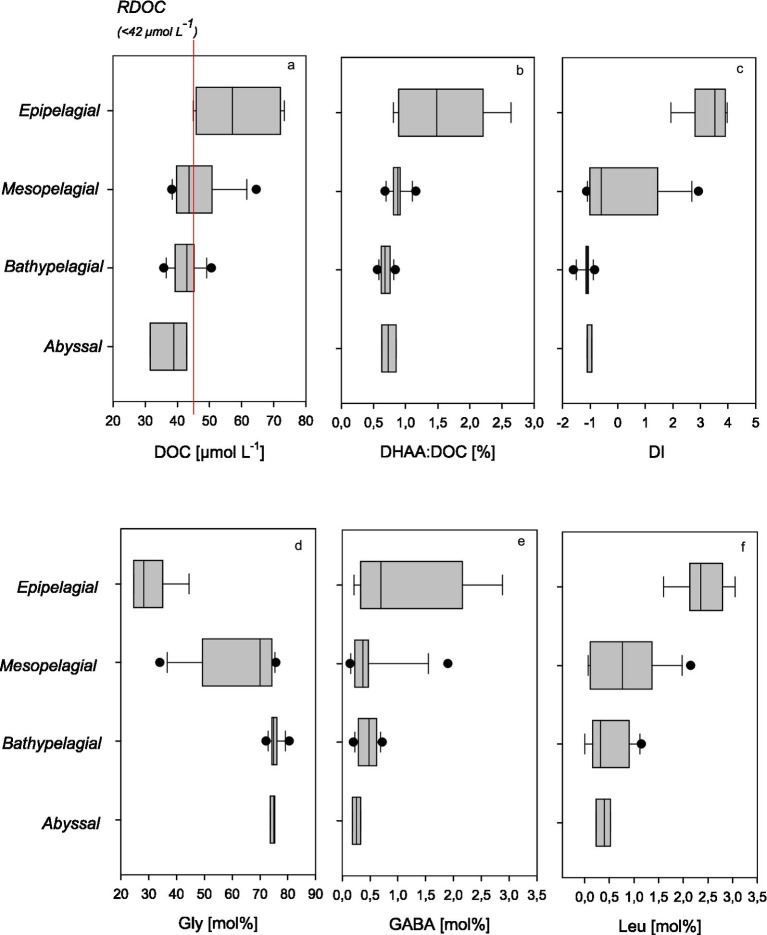
**(a–f)** Concentrations of dissolved organic carbon (DOC, **a**), the proportion of carbon contained in DHAA to DOC (DHAA:DOC, **b**), and the degradation index (DI, **c**) based on the DHAA distribution, as well as individual AAs that indicate microbial degradation of DOM, specifically glycine (Gly, **d**), GABA **(e)**, and leucine (Leu, **f**) over depth for the three stations sampled during SO288.

DOC concentrations ranged between 31.6 and 73.3 μmol L^-1,^ with the highest concentrations in the epipelagic surface samples, decreasing most pronouncedly between the epi- and mesopelagic and further to reach the lowest in the abyssal regions (Figure 4a). The ratio of DOC to POC ranged from lowest values of 5.2 in the epipelagic to 51 in the mesopelagic, declining to ~20 in the abyss. It has been suggested that RDOC in the deep sea amounts to 42 μmol L^−1^ (Hansell, 2013). Thus, most of the DOC collected in the bathypelagic zone and all DOC sampled in the abyssal zone during this study can be considered refractory. This suggests that the water column in the Humboldt Current off Chile encompasses the full spectrum of DOM lability—from highly labile compounds in the productive surface layer to ultra-refractory components persisting below 5,000 m. This broad gradient in DOC lability provides a valuable framework for examining changes in DOM composition associated with microbial diagenesis.

DHAA concentrations in our study region ranged from 0.41 to 0.10 μmol L^−1^ and decreased with depth, following a similar profile as DOC. In fresh plankton and suspended matter found in surface waters, AAs account for more than 60% of the total organic carbon. The carbon contribution of AA decreases to < 20% in sinking particles and suspended matter from subsurface waters (Wakeham and Lee, 1993). In DOM, the carbon yield of dissolved AAs has been suggested to range between 21% for DOC freshly released from plankton in incubation experiments and decreases with lability to ~ 0.7% for deep-sea RDOC (Davis and Benner, 2007). In our study, the proportion of carbon contained in DHAA (DHAA-C) relative to DOC ranged from 2.6% in the epipelagic and clearly decreased to a minimum threshold of DHAA-C of 0.6% in the abyssal, confirming the refractory nature of DOC in the bathy- and abyssal regions off Chile (Figure 4b). The highest DHAA carbon yield was similar to findings of DHAA-C:DOC of 2.4% observed for the upwelling system off Peru during an austral winter campaign (Maßmig and Engel, 2021) but considerably lower than observed in plankton incubations, suggesting that DOM in the epipelagic Humboldt current includes significant amounts of semi-labile DOC.

The measured value of 0.6% DHAA-C in RDOC suggests that AAs are not fully degraded in the marine DOM pool but persist at a stable minimum level over millennial timescales. The existence of a globally consistent residual AA-C fraction in RDOC remains puzzling and currently lacks a definitive causal explanation. One possible hypothesis is that organisms or particles smaller than the typical filtration threshold of 0.45–0.7 μm—such as bacteria, viruses, or detrital nanoparticles—contribute to the trace levels of AAs observed in deep-sea DOC. However, this alone cannot account for the widespread occurrence of similar AA-C yield values across geographically and hydrographically diverse locations. An alternative explanation is that this fraction of deep-sea AAs is ultra-refractory, meaning it is highly resistant to microbial degradation and significantly older than the ocean’s circulation turnover time.

Based on AA composition, the DI-value has often been used as an indicator for the diagenetic state of organic matter (Dauwe and Middelburg, 1998). In our samples, the DI ranged from 4 to −1.6, with the highest values observed in surface waters and the lowest in deep-water samples (Figure 4c). Although DI typically decreases with increasing diagenetic alteration, and is therefore useful for following degradation processes, the absolute DI values are influenced by the principal component loadings specific to each dataset and may not be directly comparable across studies.

Another indicator of RDOC can be derived from DHAA composition. In our study, the mol% of individual AAs varied with depth, with the most substantial changes in DHAA composition occurring between the epi- and mesopelagic zones (Figures 4d–f). The most abundant AAs in the surface waters were Gly > AsX > GlX > Ala> Thr > Ser with 24.8 ± 0.3, 19.0 ± 0.4, 12.2 ± 1.0, 11.7 ± 0.5, 9.9 ± 1.1, and 9.3 ± 0.5 mol%, respectively. Gly was also the dominant AA at all depths and the only one that proportionally increased clearly with depth (Figure 4d). In our study, Gly ranged between 24.5 mol% in surface waters and was relatively constant below the mesopelagic zone (i.e., >1,000 m depth) with a mean of 75 ± 1.8 mol%. A similar value (74 mol% Gly) was observed by Maßmig and Engel (2021) for THAA in mesopelagic waters off Peru. This suggests that 75 mol% Gly in a seawater sample may serve as an indicator value for RDOC, at least in the Humboldt upwelling system. Here, elevated Gly concentrations and distinct AAs patterns likely result from a combination of multiple processes, including high biological productivity and export, microbial diagenesis, and physical transport processes. Gly is frequently observed to become more abundant in organic matter as degradation proceeds (Lee and Cronin, 1984; Keil et al., 2000; Veuger et al., 2012) and has been used as an indicator of more diagenetically altered, refractory organic matter (Kaiser and Benner, 2009; Yamashita and Tanoue, 2003). Previous studies have shown that Gly becomes the dominant AA in marine POM as organic matter ages (Lee et al., 1983), which has also been supported by findings from anoxic pore waters (Henrichs, 1993). During periods of intense upwelling, diatoms often dominate the phytoplankton biomass, contributing to substantial amounts of sinking, diagenetically altered POC including refractory organic matrices of diatom frustules, which have high relative amounts of Gly (Hecky et al., 1973). Another potential source is the refractory bacterial cell wall component, peptidoglycan, which is also relatively rich in Gly. Furthermore, anaerobic processes within the OMZ, such as fermentation and the anaerobic oxidation of organic carbon, may enhance Gly accumulation. Srain et al. (2020) demonstrated that poor ventilation in OMZ waters off Chile favors selective Gly retention, further supporting the role of microbial and geochemical processes in shaping AA distributions in the deep ocean. In addition to *on-site* production and accumulation during microbial degradation, external sources of Gly may contribute to its distribution, such as horizontal transport of DOM enriched in Gly from nearshore sediments (Dauwe and Middelburg, 1998).

The non-proteinogenic AA *γ*-aminobutyric acid (GABA), a microbial degradation product of glutamic acid, is commonly used as an indicator of more refractory organic matter. We observed a decline of mol% of GABA below the epi- and mesopelagic zone, but no significant differences in the water column below (*p* > 0.001; *n* = 16). This suggests that GABA accumulates in the early stages of DOM degradation, but eventually degrades on longer times scales. The proteinogenic AA leucine degrades rapidly during microbial diagenesis, and has been used as a marker of labile organic matter. In our data, Leu was detectable down to abyssal depths, which may point to a supply of labile organic matter (Figure 4f).

### 3.3 Virus and prokaryotic abundance, activities, and community composition

**Figure 5:**
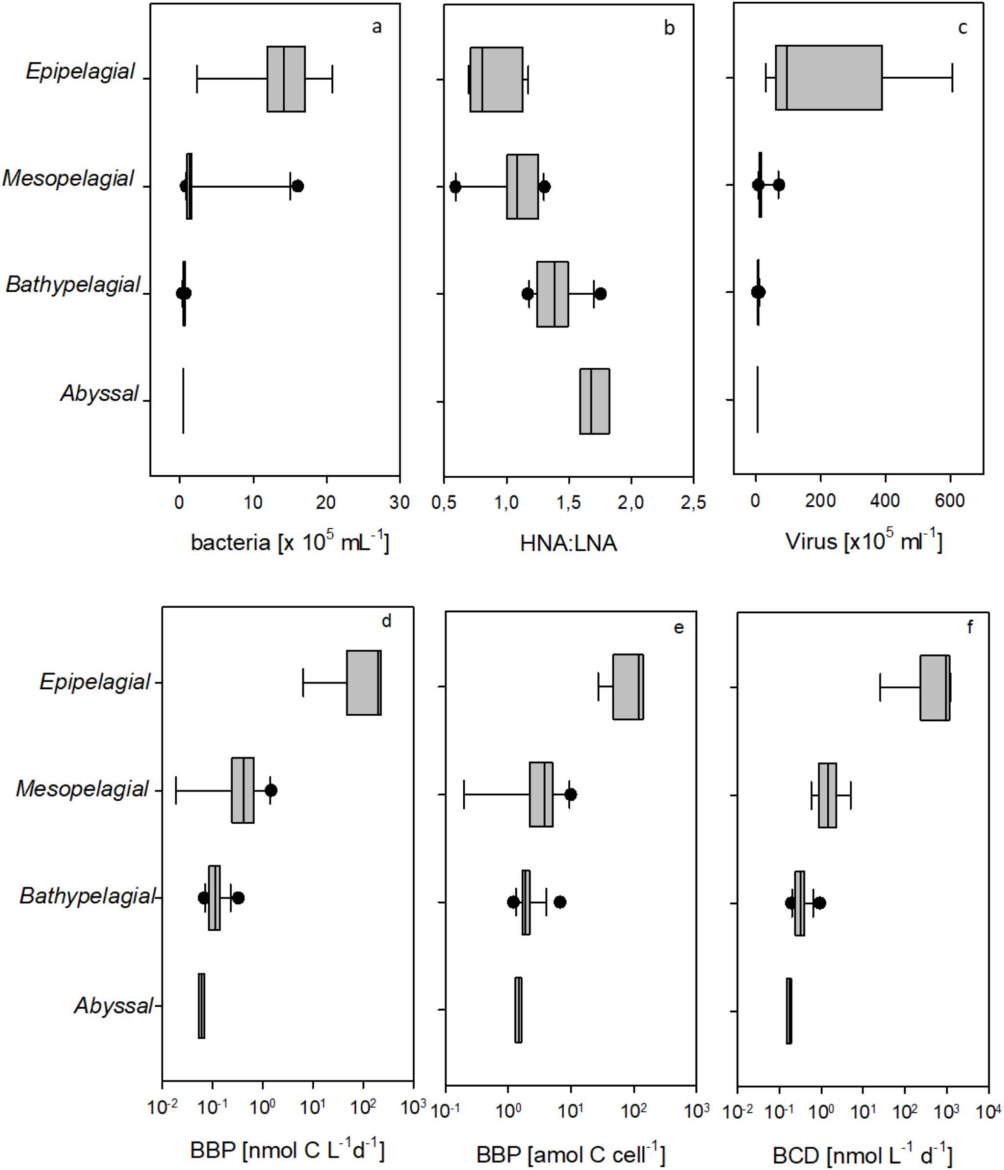
**(a–f)** Flow cytometry-based prokaryotic cell counts **(a)**, the ratio of high to low nucleic acid content prokaryotic cells (HNA:LNA, **b**), virus counts **(c)**, bacterial biomass production (BBP, **d**) based on ^3^H-leucine incorporation, BBP per bacterial cell **(e)**, and bacterial carbon demand (BCD, **f**).

Prokaryotic abundance was highest in the epipelagic layer, ranging between 2.34 × 10^5^ and 2.08 × 10^6^ cells mL^−1^ (Figure 5a). Prokaryotic abundance decreased within the mesopelagic zone by two orders of magnitude and further down to reach the lowest numbers in the deep abyss (4.11 × 10^4^ mL^−1^). Notably, the variability of prokaryotic abundance within the bathy- and abyssal samples was much lower than above, indicating low but more constant cell growth at these greater depths. Along with changes in abundance, the ratio of high to low nucleic acid content (HNA:LNA) of cells also changed (Figure 5b). While the ratio was more variable in the epi- and mesopelagic zone, the proportion of HNA cells increased with depth, peaking in the abyssopelagic zone. In general, the ratios of HNA to LNA are highly variable and influenced by numerous biogeochemical and environmental factors. HNA bacteria were found to be more abundant on particles (Lapoussière et al., 2011). While flow cytometry is not well-suited for accurately quantifying particle-associated bacteria, a portion of these cells may be released from particles during sample pre-treatment, such as sonication. Thus, a higher abundance of HNA bacteria in the deep-sea may point to higher contributions of particle associated bacteria. The increasing HNA:LNA ratio with depth is in accordance with other reports from the deep sea and may be related to the effects of hydrostatic pressure on membrane properties, compositional changes of the microbiome with depth, and cell loss via predation/viral lysis (Reinthaler et al., 2006; Bouvier et al., 2007; Van Wambeke et al., 2011; Winter et al., 2009). However, HNA cells have also been shown to have higher metabolic activity (Morán et al., 2007). For the deep sea, it was suggested that HNA cells might have an overall larger genome size, possibly reflecting a higher level of cell-specific activity or a bigger cell size in the meso- and bathypelagic ocean (Arístegui et al., 2009).

Virus abundance was high and variable within epipelagic samples, ranging between 3.0 × 10^6^ and 6.1 × 10^7^ mL^−1^, but lower throughout the deeper water column with 12 × 10^5^ ± 15 × 10^5^ mL^−1^. Virus abundance followed essentially the same abundance profile as prokaryotic cells (Figure 5c) but exceeded prokaryotic cell numbers at all depths. The lowest virus abundance was determined in the abyss with 3.4 × 10^5^ mL^−1^. The virus-to-prokaryote ratio (VPR) was highest in the epipelagic zone, with a VPR of 51, and lowest in the OMZ, with a VPR of 3.9. There was no significant relationship between VPR and depth (*r* = −0.03, *n* = 36). Our findings of virus abundance in the upwelling system off Chile are one order of magnitude higher than those reported for the subarctic and subtropical North Pacific by Hara et al. (1996), but similar to the virus abundance found in the Atlantic by Parada et al. (2007). This difference may, however, also be due to differences in the applied quantification method, as Hara et al. (1996) quantified the virus by epifluorescence microscopy, whereas Parada et al. (2007) used the same flow cytometry methods as in our study. However, in contrast to Hara et al. (1996) and our study, Parada et al. (2007) observed a clear increase in VPR ratio over depth and with decreasing prokaryote abundance.

Heterotrophic bacterial biomass production (BBP) rates were obtained only from oxygenated waters in the epipelagic, excluding the OMZ. BBP was highest in the epipelagic with 225 nmol C L^−1^ d^−1^ and continuously decreased with depth to a minimum value of 0.053 nmol C L^−1^ d^−1^ in the abyss (Figure 5d). Even excluding the epipelagic zone, BBP was significantly correlated to depth (*r* = −0.53, *n* = 25, *p* < 0.001). Normalized to cell abundance, BBP was also highest at the surface, with a maximum of 144 amol C cell^−1^ d^-1,^ and lowest in the abyss, with 1.31 amol C cell^−1^ d^−1^ (Figure 5e). Decreasing BBP with depth in the Humboldt upwelling system has also been observed off the Peruvian coast (Maßmig et al., 2020), which is also characterized by a shallow and extensive OMZ, as well as for fully oxygenated areas in the Atlantic (Baltar et al., 2009) and the equatorial Pacific (Kirchman et al., 1995). This trend indicates a shift toward low-activity communities at depth and can be attributed to the limited availability of substrates in the meso- and bathypelagic waters. BBP was significantly negatively correlated to the HNA:LNA ratio (*r* = −0.64, *n* = 25, *p* < 0.01). This observation agrees with findings from the Mediterranean Sea (Van Wambeke et al., 2011).

Pressure effects have been shown to result in higher *in situ* prokaryotic activity compared to rates measured under decompressed conditions (Tamburini et al., 2002). However, a recent meta-analysis comparing *in situ* and decompressed prokaryotic activity rates found that most deep-sea prokaryotes are piezotolerant, exhibiting similar activity regardless of pressure conditions (Amano et al., 2022). The study also revealed that a small fraction of deep-sea bacteria is piezosensitive, with activity rates up to 100 times higher under decompressed conditions. These contrasting findings highlight the need for caution when interpreting absolute activity rates of deep-sea prokaryotes.

Despite the overall low BBP, the question remains how bacteria meet their carbon demand in the deep Humboldt current, given the apparent refractory nature of DOC below the mesopelagic zone. Estimating bacterial carbon demand (BCD) requires assumptions about the bacterial growth efficiency (BGE), which in turn depends on measurements of bacterial respiration. Due to the extremely low respiration rates of deep-sea bacteria, such measurements are technically challenging, and only limited data are available for deep-sea environments. Reinthaler et al. (2006) measured prokaryotic oxygen consumption down to 4,000 m in the North Atlantic and reported a BGE of approximately 2% for the bathypelagic zone. BGE has also been estimated using the assimilation of radiolabeled substrates. For example, Tamburini et al. (2002) reported a ^14^C-glutamic acid assimilation yield of 52% at a bathypelagic site in the Mediterranean Sea and decreasing yields at shallower depths. However, because assimilation efficiency is highly dependent on the specific substrate used, extrapolating these results to general BCD values requires caution and may not reflect overall microbial activity across diverse organic substrates. For the surface ocean, it has been shown that BGE can be estimated as a function of temperature (Rivkin and Legendre, 2001). Applying the temperature dependent BGE relationship, we estimated BCD with a maximum of 0.82 ± 0.56 μmol C L^−1^ d^−1^ at the surface and an extremely low minimum of 0.0002 ± 0.0002 μmol C L^−1^ d^−1^ or 0.17 nmol C L^−1^ d^−1^ in the abyssal (Figure 5f). Over depth the BCD was significantly related to DOC concentration (*r* = 0.79, *n* = 25, *p* < 0.001) and POC concentration (*r* = 0.94, *n* = 25, *p* < 0.001). If we assume a BGE of only 2% in the bathy- and abyssopelagic zones, the estimated BCD would be higher by approximately one order of magnitude, yielding an average of 0.006 and 0.003 μmol C L^−1^ d^−1^ for the bathypelagic zone and abyssal, respectively. If we assume that RDOC was unavailable for bacterial growth, the BCD below the mesopelagic zone must be sustained by sinking particles, as suggested previously (Nagata et al., 2000; Tamburini et al., 2002).

Indeed, POC concentrations observed below the epipelagic zone were sufficiently high to support the bacterial carbon demand (BCD) for more than a year, even assuming a low BGE of 2%. To sustain this demand on longer timescales, however, POC has to be supplied by sinking particle fluxes. The supply of carbon via the biological carbon pump is often estimated from sediment trap data. For the Humboldt Current off Peru, Engel et al. (2023) quantified POC export fluxes from multiple deployments of drifting sediment traps. To obtain an estimate of the range of POC flux at 5000 m depth (*F*_z_) for our study site, we applied the Martin power-law function for particle flux attenuation (Martin et al., 1987) (Equation 1):


(1) $$F_{z}=F_{100}\times {\left(\frac{Z}{100}\right)}^{-b}$$

the lowest and highest POC flux value reported at 100 m depth (F_100_ = 51 and 412 mg C m^−2^ d^−1^) and the lowest and highest attenuation coefficient (*b* = 0.33 and −1.05) from that study. The POC consumed at 5000 m can be derived from this function using (Equation 2):


(2) $$\frac{\mathrm{dF}}{\mathrm{dz}}=-b\times F_{100}\times 100^{b}\times z^{-b-1}$$

This calculation yields a range of 0.0055 to 0.23 μmol C L^−1^ yr.^−1^ of POC being consumed at 5000 m depth. This range of values suggest that the carbon available at 5000 m could meet the BCD if BGE in the deep ocean scales with temperature, as discussed above. However, assuming a BGE of 2%, the required carbon supply would be 2.19 μmol C L^−1^ yr.^−1^, which exceeds the estimated POC flux by approximately one order of magnitude. Based on POC fluxes derived from the Martin curve and the sediment trap data off Peru, the minimum BGE required to balance carbon demand with supply would be around 8%. From an ecological view, a higher BGE would be advantageous in the deep sea, where substrates are scarce, as it would enable more efficient conversion of organic carbon into biomass. Our estimates thus support the notion that sinking particulate organic matter can sustain microbial activity at great depths, provided that microbes have relatively high growth efficiencies. Nevertheless, these considerations clearly demonstrate the sensitivity of bacterial carbon turnover estimates to assumptions about BGE and export flux attenuation.

**Figure 6:**
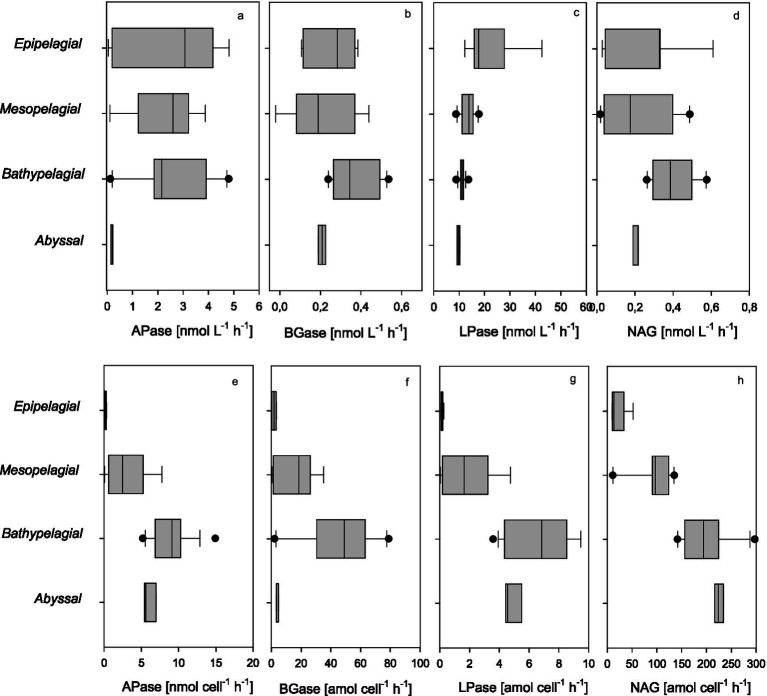
**(a–h)** Extracellular enzymatic activity (EEA) of alkaline phosphatase (APase), *β*-glucosidase (BGase), leucine aminopeptidase (LPase), and β-N-acetyl-glucosaminidase (NAG) for bulk **(a–d)** and cell-specific **(e–h)** measurements.

To investigate the potential microbial degradation of two major components of organic matter, protein and carbohydrates, we measured the rates of extracellular enzymatic activity (EEA) such as alkaline phosphatase (APase), *β*-glucosidase (BGase), leucine aminopeptidase (LAPase) and β-N-acetyl-glucosaminidase (NAG) (Figures 6a–h). Bulk EEA profiles of these enzymes varied across the water column, with distinct gradients from epipelagic to abyssopelagic zones and from coastal to offshore stations, largely attributed to enhanced nutrient availability associated with upwelling dynamics (Figures 6a–d). Bulk LAPase hydrolysis rates peaked at 20.5 ± 6.4 nmol L^−1^ h^−1^ in the epipelagic and decreased ~2-fold with depth. In contrast, BGase activity increased up to ~4.5-fold with depth, reaching the highest rates at 0.5 ± 0.03 nmol L^−1^ h^−1^ in the bathypelagic. Bulk LAPase rates shown in this study (10.87 to 20.71 nmol L^−1^ h^−1^) were within the range from a study in the eastern tropical South Pacific off Peru (9 to 158 nmol L^−1^ h^−1^; Maßmig et al., 2020). Although similar LAPase trends have been reported for the western subtropical North Atlantic (Baltar et al., 2009), compared to other studies, LAPase rates obtained from the Humboldt upwelling system were several orders of magnitude higher than those reported from the Mediterranean Sea down to 2000 m depth (0.034 to 2.77 and 0.23 to 1.78 nmol L^−1^ h^−1^; Zaccone et al., 2003; Tamburini et al., 2002), and roughly twice as high as in the subtropical North Atlantic (0.6 to 9.2 nmol L^−1^ h^−1^; Baltar et al., 2009), the Tyrrhenian Sea down to 3,500 m depth (0.51 to 8.6 nmol L^−1^ h^−1^; Tamburini et al., 2009), and the Indian Ocean (6 to 15 nmol L^−1^ h^−1^; Hoppe and Ullrich, 1999). The intensified upwelling by La Niña and the associated increase in phytoplankton biomass may explain the ~1.5 times higher LAPase rates in the Humboldt upwelling system. The potential bulk BGase activity reported here from the Humboldt upwelling system was on average three-fold lower compared to suboxic waters (1.6 ± 1.5 nmol L^−1^ h^−1^) and the oxycline (1.2 ± 0.6 nmol L^−1^ h^−1^) in eastern tropical South Pacific off Peru (Maßmig et al., 2020), but within the range of those reported in various oceanic regions such as the subtropical North Atlantic (0.1 to 3.9 nmol L^−1^ h^−1^; Baltar et al., 2009), the Baltic Sea (0.2 to 2 nmol L^−1^ h^−1^; Nausch et al., 1998), and the Arabian Sea (0.27 to 1.18 nmol L^−1^ h^−1^; Hoppe and Ullrich, 1999).

Cell-specific potential hydrolytic activity generally increased with depth (Figures 6e–h). On average, cell-specific BGase increased strongly with depth, from 0.14 amol cell^−1^ h^−1^ in the epipelagic to 6.7 amol cell^−1^ h^−1^ in bathypelagic and cell-specific LAPase from 17.9 amol cell^−1^ h^−1^ to 225 amol cell^−1^ h^−1^ in the abyssopelagic. The increasing cell-specific enzyme activities align well with the relative increase in HNA cells, supporting the notion that microbial activity in the dark ocean occurs mainly on POM and colloidal material with generally higher fractions of dissolved EEA compared to free-living microbes (Baltar et al., 2010; Baltar et al., 2009).

**Figure 7:**
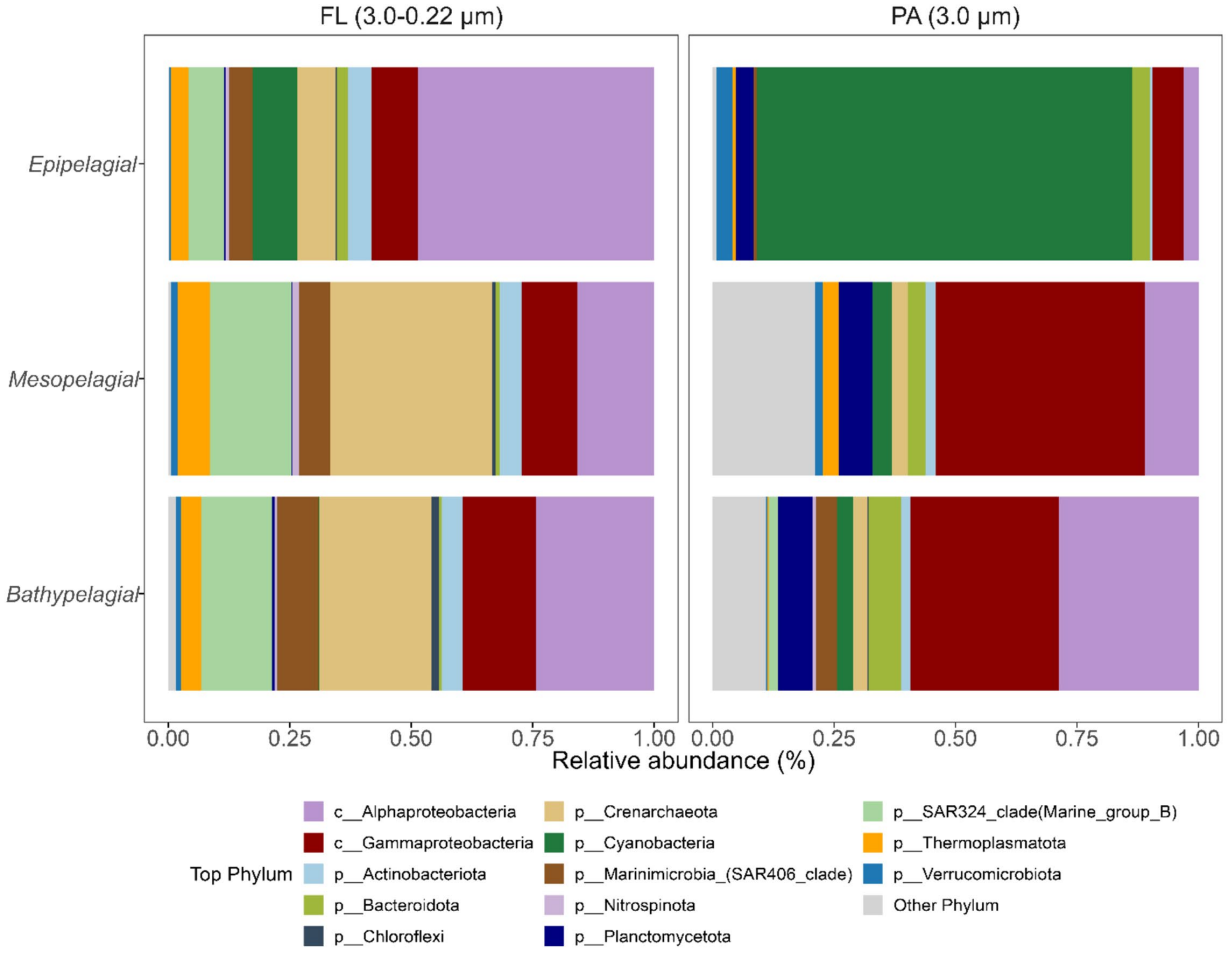
Microbiome community composition derived from 16S miTAG analysis. Relative abundance of prokaryotes (phylum level) in each size fraction grouped into pelagic zones.

Microbial community structure differed substantially between free-living (FL) and particle-associated (PA) lifestyles and depth layers (Figure 7). In the surface layer, the FL microbiome composition showed high relative proportions of Alphaproteobacteria (62%) and Cyanobacteria (16.3%). However, with depth, microbiome composition changed profoundly; for instance, the relative abundances of *Crenarchaeota* (~30%), SAR324 clade (~16%), *Marinimicrobia* (~7%), and *Chloroflexi* (~1%) increased toward meso- and bathypelagic zones. In contrast, the PA surface microbiome was characterized by a high proportion of Cyanobacteria due to the larger cell size, accounting for ~89% of relative abundance, and *Verrucomicrobiota* (3.8%). Notably, at 100 m depth, PA microbiome composition shifted to high proportions of Gammaproteobacteria (38.5%) and Planctomycetota (22.1%). In the mesopelagic zone, the relative abundance of Gammaproteobacteria peaked at 42.5% relative abundance along with the maximum relative abundance of *Thermoplasmatota* at 750 m depth (7.4%). The relative proportion of Alphaproteobacteria in PA increased with depth, reaching 11.3 and 27.1% in meso- and bathypelagic zones. Bacteroidota contributed relative abundances of ~5.3, 3.2, and 7.7% to the PA microbiome in the epi-, meso-, and bathypelagic zones, respectively, but were not detected at a depth of 750 m (Figure 7).

The depth-related changes in microbiome structure have been well documented and are associated with changes in both abiotic and biotic variables. It has been shown that genes encoding secretory processes, i.e., dissolved enzymes, consistently increase (abundance, percentage, and diversity) from epipelagic to bathypelagic waters, and that especially Bacteroidetes and Gammaproteobacteria contribute to the pool of secretory carbohydrate-active enzymes (CAZymes) and peptidases in the bathypelagic, suggesting that prokaryotic metabolism is mediated mainly by particle-associated prokaryotes such as Bacteroidetes and Gammaproteobacteria that seem to have a preference for POM and fast-sinking particles (Zhao et al., 2020).

## 4 Conclusion

Overall, the results of this study in the Humboldt upwelling system suggests that below the mesopelagic zone, DOC appears to be refractory in nature and characterized by very low DOC concentrations (<42 μmol C L^−1^), AA yields below 1% (~ 0.6%), and a relatively high proportion of Gly (~75 mol% DHAA). Given these constraints of labile to semi-labile substrates and the fact that microbial activities (e.g., cell-specific EEA) and the proportion of high nucleic acid (HNA) cells increased with depth, it is likely that deep-sea microbial activity is to a large extent supported by organic carbon supplied by sinking particles. These patterns are likely a result of a combination of processes: (i) high biological productivity and export as indicated by relatively high Chl *a* concentration, relative abundance of Cyanobacteria and bacterial taxa known for association with particles such as Bacteroidetes in PA (3.0 μm pore size fraction), also in the meso- and bathypelagic zones. (ii) Microbial diagenesis as indicated by the amino acid degradation index, suggesting increasing DOM degradation with depth together with relatively high LAPase activities, suggesting pronounced levels of protein degradation, and (iii) Physical transport processes associated with periods of intense upwelling promoting phytoplankton growth, typically with a dominance of diatoms. Diatom frustules consist of high proportions of Gly and can significantly contribute to the export of diagenetically altered POC. (iv) Unique hydrography in the Humboldt upwelling system, affecting the ventilation in OMZ waters off Chile, likely contributes to the selective enrichment of Gly. Consequently, our study demonstrates that a combination of microbial, geochemical, and physical processes shapes microbial organic matter diagenesis in the Humboldt upwelling system and highlights the importance of sinking particles to fuel deep-sea microbes.

## Data Availability

The original contributions presented in the study are publicly available. This data can be found here: PANGEA, https://doi.pangaea.de/10.1594/PANGAEA.987839.
