# Supplementary material for: Microbial diagenesis of dissolved organic matter from the ocean’s surface to abyssal depths: a case study in the Humboldt upwelling system
Source: Front Microbiol. 2025 Dec 16;16:1677097. doi: 10.3389/fmicb.2025.1677097 (PMC12753110; doi:10.3389/fmicb.2025.1677097)
Supplement: Supplementary file 1 [file Data_Sheet_1.pdf]

1 Supplementary Material

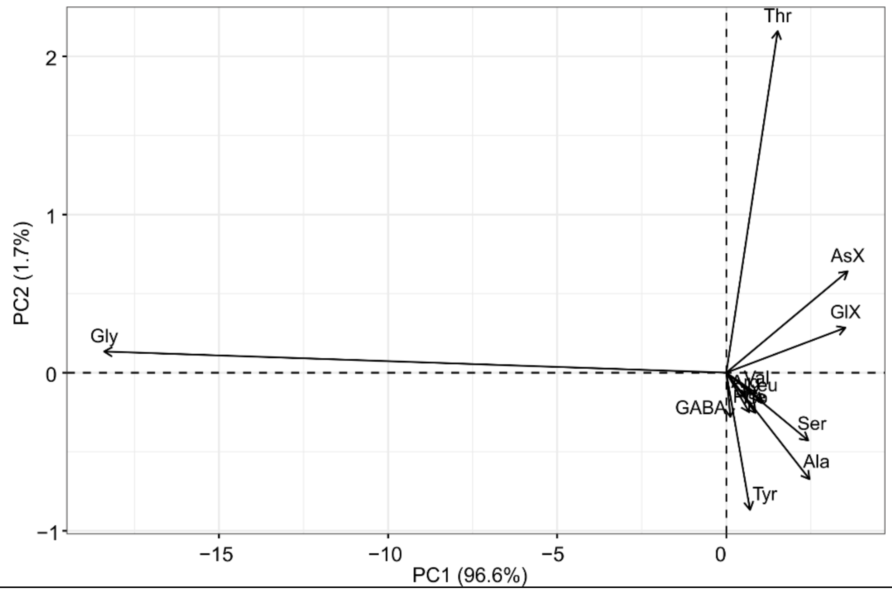

PC1	0.18	0.180	0.123	-0.94	0.077	0.028	0.125	0.006	0.035	0.045	0.044	0.034	0.054
factor	357	227	933	38	709	767	899	081	913	954	02	637	462
loading													

Figure S1: Principal component analysis (PCA) of DHAA and factor loadings used for calculation of the degradation index (DI) according to Dauwe (1999).

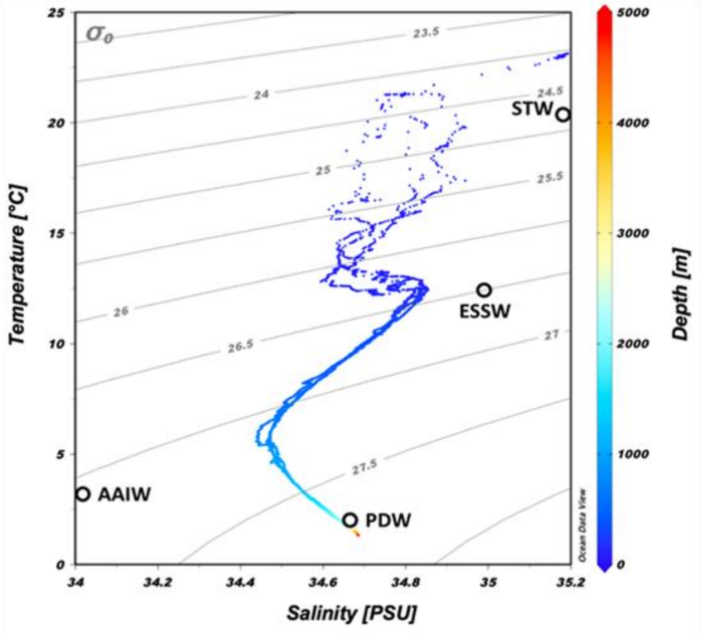

Figure S2: Water masses encountered during the SO288 expedition off Chile. Reference water masses: Subtropical water (STW), equatorial sub-surface water (ESSW), Pacific deep water (PDW) and Antarctic intermediate water (AAIW) according to Silva et al. (2009) and Rosmann (2024).

<u>Reference:</u>

Silva, N., Rojas, N., & Fedele, A. (2009). Water masses in the Humboldt Current System: Properties, distribution, and the nitrate deficit as a chemical water mass tracer for Equatorial Subsurface Water off Chile. *Deep Sea Research Part II: Topical Studies in Oceanography*, *56*(16), 1004-1020.

Rosmann, Mara (2024). Transparent Exopolymer Particles from the surface to the deep ocean: High resolution full-depth profiles in the Humboldt Upwelling System off Central Chile. MSc Thesis, University of Kiel.

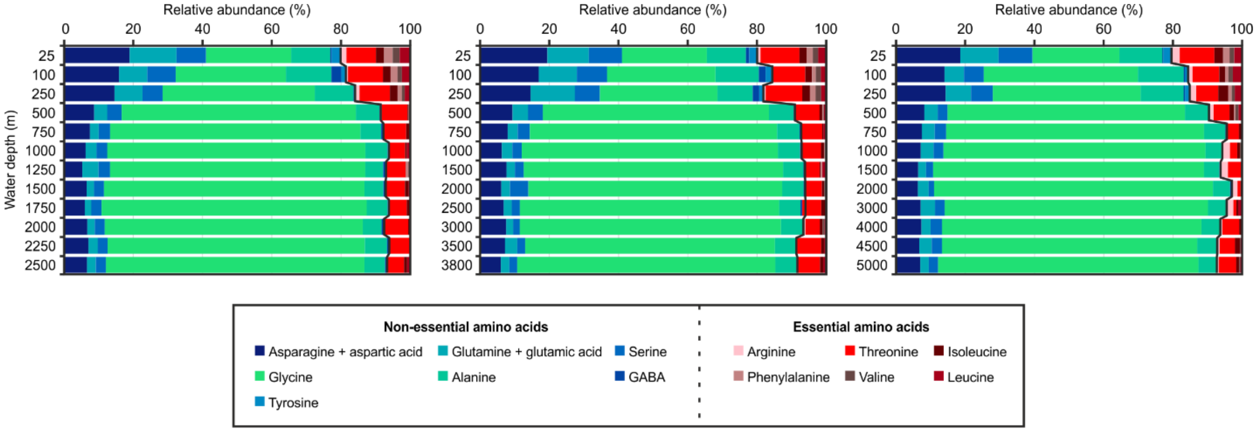

Fig. S3: Changes in DHAA composition over depth at the three sampling sites.
